# The number of reduced alignments between two DNA sequences

**DOI:** 10.1186/1471-2105-15-94

**Published:** 2014-04-01

**Authors:** Helena Andrade, Iván Area, Juan J Nieto, Ángela Torres

**Affiliations:** 1 Departamento de Análise Matemática, Facultade de Matemáticas, Universidade de Santiago de Compostela, 15782 Santiago de Compostela, Spain; 2 Departamento de Matemática Aplicada II, E.E. Telecomunicación, Universidade de Vigo, 36310 Vigo, Spain; 3 Faculty of Science, King Abdulaziz University, P.O. Box 80203, 21589 Jeddah, Saudi Arabia; 4 Departamento de Psiquiatría, Radioloxía e Saúde Pública, Facultade de Medicina, Universidade de Santiago de Compostela, 15782 Santiago de Compostela, Spain

**Keywords:** DNA sequence, Alignment, Difference equation

## Abstract

**Background:**

In this study we consider DNA sequences as mathematical strings. Total and reduced alignments between two DNA sequences have been considered in the literature to measure their similarity. Results for explicit representations of some alignments have been already obtained.

**Results:**

We present exact, explicit and computable formulas for the number of different possible alignments between two DNA sequences and a new formula for a class of reduced alignments.

**Conclusions:**

A unified approach for a wide class of alignments between two DNA sequences has been provided. The formula is computable and, if complemented by software development, will provide a deeper insight into the theory of sequence alignment and give rise to new comparison methods.

**AMS Subject Classification:**

Primary 92B05, 33C20, secondary 39A14, 65Q30

## Background

Let us consider a DNA sequence as a mathematical string

$$x=\left(x_{1},x_{2},\ldots ,x_{n}\right),$$

where *x*_*i*_∈{*A*,*G*,*C*,*T*} is one of the four nucleotides, *i*=1,2,…,*n*, i.e. *A* denotes adenine, *C* cytosine, *G* guanine and *T* thymine. In these conditions, the sequence *x* is of length *n*.

Our main goal is to compare the sequence *x* with another DNA sequence

$$y=\left(y_{1},y_{2},\ldots ,y_{m}\right),$$

to measure the similarity between both strings and also to determine their residue-residue correspondences.

Sequence comparison and alignment is a central and crucial tool in molecular biology. For example, Pairwise Sequence Alignment is used to identify regions of similarity that may indicate functional, structural and/or evolutionary relationships between two biological sequences (protein or nucleic acid) [1].

For some recent developments and directions we refer the reader to [2-7] and [8] for a general review of different alignments methods.

To align the sequences *CGT* and *ACTT*, one can use EMBOSS Needle for nucleotide sequence [9] that creates an optimal global alignment of the two sequences using the Needleman-Wunsch algorithm to get

$$\begin{array}{ccccccc} \text{EMBOSS-001} & \;\;1 & \;\;\text{-} & \;\;C & \;\;G & \;\;T & \;\;3\\ \;\; & \;\; & \;\; & \;\;|\;\; & \;\;\cdot & \;\;|\\ \text{EMBOSS-001} & \;\;1 & \;\;A & \;\;C & \;\;T & \;\;T & \;\;4 \end{array}$$

Following Lesk [10], in order to compare the amino acids appearing at their corresponding positions in two sequences, theirs correspondences must be assigned and a sequence alignment is the identification of residue-residue correspondence. For some references on sequence alignment we refer the reader to [10-16].

To compare two sequences, there exist mainly three different possibilities leading to three different numbers of total alignments [10,11,13]: 

1. The total number of alignments denoted by *f*(*n*,*m*) that was solved in [13].

2. A gap in a sequence is followed by another gap in the other sequence as in Alignments 1 and 2 for the sequences *x*=*CGT* and *y*=*ACTT* (see Tables 1 and 2 below) Considering the two alignments as equivalents to the Alignment 3 (see Table 3) without gap in those positions, we have the number of reduced alignments denoted by *h*(*n*,*m*), and obviously *h*(*n*,*m*)<*f*(*n*,*m*). This case has been solved in [11], and we give here another representation in terms of hypergeometric series.

3. In the interesting case that the alignments 1 and 2 are equivalent, but different from alignment 3 we have a number or reduced alignments *g*(*n*,*m*) where *h*(*n*,*m*)<*g*(*n*,*m*)<*f*(*n*,*m*). This last case is new and we present an explicit formula for *g*.

**Table 1:** Alignment 1

*C*	*G*	–	*T*	–
*A*	–	*C*	*T*	*T*

**Table 2:** Alignment 2

*C*	–	*G*	*T*	–
*A*	*C*	–	*T*	*T*

**Table 3:** Alignment 3

*C*	*G*	*T*	–
*A*	*C*	*T*	*T*

## Results and discussion

## Number of ***f(x,y)*** alignments

The total number of alignments *f*(*x*,*y*) satisfies the following recurrence relation [13]

f(n,m)=f(n−1,m)+f(n,m−1)+f(n−1,m−1),

with initial conditions *f*(*n*,0)=*f*(0,*m*)=1 for *n*,*m*=1,2,3,…. The solution of the above partial difference equation is given by

f(n,m)=∑k=0min{n,m}2kmknk,

(see formula (10) in [13]) and the generating function [17,18] is

$$F(x,y)=-\frac{1}{\text{xy}+x+y-1}.$$

Therefore the coefficients *f*(*n*,*m*) in the expansion

$$F(x,y)=\overset{\infty }{\underset{n=0}{\sum }}\overset{\infty }{\underset{m=0}{\sum }}f(n,m)x^{n}y^{m}$$

are given in terms of a hypergeometric series by

$$f(n,m)={\;}_{2}F_{1}(-m,-n;1;2).$$

This relation seems to be new in this form. Here, the generalized hypergeometric series is defined as (see e.g. [19, Chapter 16])

$${\;}_{p}F_{q}\left(a_{1},\ldots ,a_{p};b_{1},\ldots ,b_{q};d\right)=\overset{\infty }{\underset{i=0}{\sum }}\frac{{\left(a_{1}\right)}_{k}{\left(a_{2}\right)}_{k}\cdots {\left(a_{p}\right)}_{k}}{k!{\left(b_{1}\right)}_{k}{\left(b_{2}\right)}_{k}\cdots {\left(b_{q}\right)}_{k}}d^{k},$$

and (*A*)_*k*_=*A*(*A*+1)⋯(*A*+*n*−1), with (*A*)_0_=1, denotes the Pochhammer’s symbol. It is assumed that *b*_*j*_≠−*k* in order to avoid singularities in the denominators. If one of the parameters *a*_*j*_ equals to a negative integer, then the sum becomes a terminating series.

## Number of ***h(x,y)*** alignments

In this case, the recurrence relation for the *h*(*n*,*m*) coefficients is [11]

$$\begin{array}{ll} h(n,m) & =h(n-1,m)+h(n,m-1)-h(n-2,m-2),\\ & \;n,m\geq 2, \end{array}$$

with initial conditions *h*(*n*,0)=*h*(0,*m*)=1. Therefore, the generating function [17,18] is

$$H(x,y)=\frac{1-\text{xy}}{x^{2}y^{2}-x-y+1},$$

and the coefficients in the expansion

$$H(x,y)=\overset{\infty }{\underset{n=0}{\sum }}\overset{\infty }{\underset{m=0}{\sum }}h(n,m)x^{n}y^{m}$$

are given by

$$\begin{array}{ll} h(n,m) & =\overset{A}{\underset{i=0}{\sum }}\frac{{\left(-1\right)}^{i}(-3i+m+n)!}{i!(m-2i)!(n-2i)!}\\ & \;-\overset{B}{\underset{i=0}{\sum }}\frac{{\left(-1\right)}^{i}(-3i+m+n-2)!}{i!(-2i+m-1)!(-2i+n-1)!}, \end{array}$$

where

$$\begin{array}{l} A=\min\left\{\left[\frac{n}{2}\right],\left[\frac{m}{2}\right]\right\},\\ B=\min\left\{\left[\frac{n-1}{2}\right],\left[\frac{m-1}{2}\right]\right\}. \end{array}$$

The above coefficients can be written in terms of (terminating) hypergeometric series as

$$\begin{array}{ll} \frac{(m+n)!}{m!n!} & \;4F3\left(\begin{array}{ll} \begin{array}{l} \frac{1-m}{2},-\frac{m}{2},\frac{1-n}{2},-\frac{n}{2}\\ \frac{-m-n}{3},\frac{-m-n+1}{3},\frac{-m-n+2}{3} \end{array} & \frac{16}{27} \end{array}\right)\\ & \;-\frac{(m+n-2)!}{(m-1)!(n-1)!}\\ & \;4F3\left(\begin{array}{ll} \begin{array}{l} \frac{1-m}{2},1-\frac{m}{2},\frac{1-n}{2},1-\frac{n}{2}\\ \frac{-m-n+2}{3},\frac{-m-n+3}{3},\frac{-m-n+4}{3} \end{array} & \frac{16}{27} \end{array}\right)\;. \end{array}$$

## Number of ***g(x,y)*** alignments

As indicated before, the main aim of this paper is to give an explicit representation in this case. The recurrence relation for the *g*(*n*,*m*) coefficients is [11]

$$\begin{array}{ll} g(n,m) & =g(n-1,m-1)+g(n-1,m)+g(n,m-1)\\ & \;-2g(n-2,m-2),\;n,m\geq 2, \end{array}$$

with initial conditions *g*(*n*,0)=*g*(*m*,0)=1. Thus, the generating function [17,18] is

(1) $$G(x,y)=\frac{1-\text{xy}}{2x^{2}y^{2}-\text{xy}-x-y+1}.$$

### 

Theorem 1. *The coefficientsα*_*n*,*m*_*in the expansion*

(2) $$G(x,y)=\frac{1-\text{xy}}{2x^{2}y^{2}-\text{xy}-x-y+1}=\overset{\infty }{\underset{n=0}{\sum }}\overset{\infty }{\underset{m=0}{\sum }}\alpha_{n,m}x^{n}y^{m}$$

are explicitly given by

(3) $$\begin{array}{ll} \alpha_{n,m} & =\left(\overset{n+m}{\underset{i=U(n,m)}{\sum }}\overset{B(i,n,m)}{\underset{j=A(i,n,m)}{\sum }}\beta_{i,j,n,m}\right)\\ & \;-\left(\overset{n+m-2}{\underset{i=U(n,m)-1}{\sum }}\overset{D(i,n,m)}{\underset{j=C(i,n,m)}{\sum }}\gamma_{i,j,n,m}\right), \end{array}$$

where

(4) $$\begin{array}{c} \beta_{i,j,n,m}=\frac{{\left(-1\right)}^{i-j}2^{i-j}\;i!}{(i\;-\;j)!\;(2i\;-\;j\;-\;m)!\;(2i-j-n)!\;(3j-4i+m+n)!}, \end{array}$$

(5) $$\begin{array}{c} \gamma_{i,j,n,m}\;=\;\frac{{\left(-1\right)}^{i-j}2^{i-j}\;i!}{(i\;-\;j)!(2i\;-\;j\;-\;m\;+\;1)!\;(2i\;-\;j\;-\;n\;+\;1)!\;(3j\;-\;4i\;+\;m\;+\;n\;-\;2)!}, \end{array}$$

(6) $$\;A(i,n,m)=\max\left\{0,\left[\frac{4i-m-n}{3}\right]\right\},$$

(7) $$\;B(i,n,m)=\min\left\{i,2i-m,2i-n,\left[\frac{4i-n-m}{2}\right]\right\},$$

(8) $$\;C(i,n,m)=\max\left\{0,\left[\frac{4i-m-n-2}{3}\right]\right\},$$

(9) $$\begin{array}{ll} \;D(i,n,m) & =\min\left\{i,2i-m+1,2i-n+1,\right)\\ & \;\left(\left[\frac{4i-n-m+2}{2}\right]\right\}, \end{array}$$

(10) $$\;U(n,m)=\left\{\;\begin{array}{ll} m-\left[n/2\right], & \;\;n\leq m,\\ \left[(m+1)/2\right]+n-m, & \;\;n\geq m, \end{array}\right)$$

and [ *x*] denotes the integer part of *x*.

### 

*Proof*. If we expand,

(11) G(x,y)=(1−xy)∑i=0∞x+y+xy−2x2y2i=(1−xy)×∑i=0∞∑j=0i∑k=0j∑s=0k(−1)i−j2i−jijjkksy2i−j−sx2i−j−k+s,

we have two summands to be computed, namely

(12) ∑i=0∞∑j=0i∑k=0j∑s=0k(−1)i−j2i−jijjkksy2i−j−sx2i−j−k+s

(13) −xy∑i=0∞∑j=0i∑k=0j∑s=0k(−1)i−j2i−jijjkksy2i−j−sx2i−j−k+s.

In order to compute the first sum (12) let us introduce

(14) m=2i−j−s,n=2i−j−k+s.

Therefore, the summation to be done reads as

∑n=0∞∑m=0∞∑i=UV∑j=AB(−1)i−j2i−jijj4i−2j−m−n4i−2j−m−n2i−j−mxnym

where *U*, *V*, *A* and *B* must be computed in terms of the initial indices.

The product of binomials can be simplified to

$$\frac{i!}{(i-j)!\;(2i-j-m)!\;(2i-j-n)!\;(3j-4i+m+n)!}.$$

Thus,

$$\;\begin{array}{c} i\geq 0,\;j\geq 0,\;4i-2j-m-n\geq 0,\;4i-2j-m\\ -n\geq 0,\;2i-j-m\geq 0,\;i-j\geq 0,\;2i-j\\ -m\geq 0,\;2i-j-n\geq 0,\;3j-4i+m+n\geq 0, \end{array}$$

and then

$$\begin{array}{lll} A(i,n,m) & =A=\max\left\{0,\left[\frac{4i-m-n}{3}\right]\right\}\leq j\; & \;\\ & \leq \min\left\{i,2i-m,2i-n,\left[\frac{4i-n-m}{2}\right]\right\}\; & \;\\ & =B(i,n,m)=\mathrm{B.}\; & \; \end{array}$$

Finally, the summation reads as

$$\begin{array}{l} \overset{\infty }{\underset{n=0}{\sum }}\overset{\infty }{\underset{m=0}{\sum }}\left(\overset{n+m}{\underset{i=U(n,m)}{\sum }}\overset{B}{\underset{j=A}{\sum }}\right)\\ \;\left(\frac{{\left(-1\right)}^{i-j}2^{i-j}\;i!}{(i-j)!\;(2i\;-\;j\;-\;m)!\;(2i-j-n)!\;(3j\;-\;4i\;+\;m\;+\;n)!}\right)x^{n}y^{m}, \end{array}$$

where

$$U(n,m)=\left\{\;\begin{array}{ll} m-\left[n/2\right], & \;\;n\leq m,\\ \left[(m+1)/2\right]+n-m, & \;\;n\geq \mathrm{m.} \end{array}\right)$$

A similar work with the second summand (13) leads to the final result.

Some numerical values are *g*(10,10)=2003204, *g*(50,50)=2.71972×10^34^, *g*(100,100)=7.55997×10^69^, and we note that *g*(*n*,*n*)>10^80^ for *n*≥115. This last inequality is relevant since 10^80^ is an estimation of the number of protons of our universe [13].

## Conclusions

A unified approach for a wide class of alignments between two DNA sequences has been provided. We conclude also that our approach gives an explicit formula filling a gap in the theory of sequence alignment. The formula is computable and, if complemented by software development, will provide a deeper insight into the theory of sequence alignment and give rise to new comparison methods. It may be used also, in the future, to get explicit formulas and compute the number of total, reduced, and effective alignments for multiple sequences.

## Methods

We have performed a number of numerical computations to compare our formulae and Mathematica®; [20] command Coefficient for the series expansion of (1), on a MacBook Pro featuring a 45 nm “Penryn” 2.66 GHz Intel “Core 2 Duo” processor (P8800), with two independent processor “cores” on a single silicon chip, 8 GB of 1066 MHz DDR3 SDRAM (PC3-8500). We would like to mention that our approach is amazingly fast, since e.g. *g*(100,100) is computed by using Mathematica®; in 0.125165 seconds by using the new formulas presented in this paper, while the use of Mathematica®; command Coefficient needs 99.167659 seconds.

## Competing interests

The authors declare that they have no competing interests.

## Authors’ contributions

Each of the authors HA, IA, JJN and AT, contributed to each part of this study equally and read and approved the final version of the manuscript.
